# The 2G allele of promoter region of Matrix metalloproteinase-1 as an essential pre-condition for the early onset of oral squamous cell carcinoma

**DOI:** 10.1186/1471-2407-7-187

**Published:** 2007-10-05

**Authors:** Rishiho Nishizawa, Masaki Nagata, Arhab A Noman, Nobutaka Kitamura, Hajime Fujita, Hideyuki Hoshina, Takehiko Kubota, Manami Itagaki, Susumu Shingaki, Makoto Ohnishi, Hiroshi Kurita, Kouji Katsura, Chikara Saito, Hiromasa Yoshie, Ritsuo Takagi

**Affiliations:** 1 Department of Dentistry and Oral Surgery, Shinshu University School of Medicine, Asahi 3-1-1, Matsumoto 390-8621, Japan; 2 Division of Oral and Maxillofacial Surgery, Niigata University Graduate School of Medical and Dental Sciences, Gakkocho-dori 2-5274, Niigata 951-8514, Japan; 3 Division of Periodontology, Niigata University Graduate School of Medical and Dental Sciences, Gakkocho-dori 2-5274, Niigata 951-8514, Japan; 4 Division of Reconstructive Surgery for Oral and Maxillofacial Region, Niigata University Graduate School of Medical and Dental Sciences, Gakkocho-dori 2-5274, Niigata 951-8514, Japan; 5 Division of Dental Clinic and Oral Surgery, Nagaoka Red Cross Hospital, Terashimamachi 297-1, Nagaoka 940-2085, Japan; 6 Division of Oral and Maxillofacial Radiology, Niigata University Graduate School of Medical and Dental Sciences, Gakkocho-dori 2-5274, Niigata 951-8514, Japan

## Abstract

**Background:**

Matrix metalloproteinase (*MMP*) is known to be involved in the initial and progressive stages of cancer development, and in the aggressive phenotypes of cancer. This study examines the association of single nucleotide polymorphisms in promoter regions of *MMP-1 *and *MMP-3 *with susceptibility to oral squamous cell carcinoma (OSCC).

**Methods:**

We compared 170 Japanese OSCC cases and 164 healthy controls for genotypes of *MMP-1 *and *MMP-3*.

**Results:**

The frequency of the *MMP-1 *2G allele was higher and that of the 1G homozygote was lower in the OSCC cases (*p *= 0.034). A multivariate logistic regression analysis revealed that subjects who were 45 years old or older had a significantly increased (2.47-fold) risk of OSCC (95%CI 1.47–4.14, *p *= 0.0006), and those carrying the *MMP-1 *2G allele had a 2.30-fold risk (95%CI 1.15–4.58, *p *= 0.018), indicating independent involvement of these factors in OSCC. One of the key discoveries of this research is the apparent reduction of the *MMP-1 *1G/1G and 1G/2G genotype distributions among the early onset OSCC cases under the ages of 45 years. It should be noted that the tongue was the primary site in 86.2% of these early onset cases. This could suggest the specific carcinogenic mechanisms, i.e. specific carcinogenic stimulations and/or genetic factors in the tongue.

**Conclusion:**

Since the 2G allele is a majority of the *MMP-1 *genotype in the general population, it seems to act as a genetic pre-condition in OSCC development. However this report suggests a crucial impact of the *MMP-1 *2G allele in the early onset OSCC.

## Background

Matrix metalloproteinase *(MMP)-1 (Collagenase-1) *is a major proteinase of the *MMP *family that specifically degrades type I collagen, which is a major component of the extracellular matrix (ECM), as well as other fibrillar collagens of types II, III, V and IX [1,2]. *MMP-3 *(Stromelysine-1) is responsible for degradation of type IV collagen, which forms the basement membrane, and collagen V, IX, X [3]. *MMP-3 *also has a role in activation of *proMMP-1 *in tumor tissue into the active form of *MMP-1 *[4]. Gene expression data in our previous report demonstrated enhanced expression of *MMP *family genes in OSCC tissues, and suggested correlation of high expression levels of *MMP-1 *and *MMP-3 *with aggressive behavior, such as metastasis, and clinical prognosis [5]. Similar correlations have been reported in pharyngeal, colon and various other tumor types [6-8]. Colocalization of *MMP-1 *and *MMP-3 *with destruction of ECM in the invasive front of cancer tissue suggests a direct role in cancer invasion [9].

It has also been shown that a function of *MMPs *affects susceptibility to different kinds of carcinoma. To date, polymorphisms of the promoter domain have been described in *MMP-1, -3, -9, -12 *to influence the expression level of the genes [10]. A 2G type of single nucleotide polymorphism (SNP) at -1607 bp site in the promoter domain of *MMP-1 *creates a sequence, 5'-GGA-3,' that is the core recognition sequence of the binding site for Ets family transcription factors. The 2G type promoter results in higher transcription activity of the *MMP-1 *gene than does a 1G type promoter [11]. A 5A type promoter at -1171 bp site of *MMP-3 *is also known to have a twofold higher transcriptional activity in vitro than that of a 6A type [12]. It has been documented that the 2G type SNP of *MMP-1 *confers increased susceptibility to colorectal [13], ovarian [14], lung [15], endometrial [16], renal cell [17] and head and neck [18] cancers; and the 5A type SNP of *MMP-3 *is associated with an increased susceptibility to breast cancer [19].

An in situ hybridization study revealed that *MMP-1 *expression in normal oral mucosa is controlled at a low level, whereas a remarkably elevated expression level is observed in cases of oral epithelial dysplasia, which becomes even higher in cases of OSCC [20]. It has also been documented that cases of oral epithelial dysplasia showing high expression level of *MMP-1 *developed into OSCC at higher frequency than cases with low expression of *MMP-1 *[21]. Taking all the observations noted above into consideration, it seems likely that *MMP*s may serve as key factors in all stages of the OSCC progression from carcinogenesis in the early precancerous condition to the advanced invasive and metastatic phases.

The purpose of this study is to document the effect of genomic polymorphisms of *MMP *genes in the development of OSCC. We compared genotype distribution in the promoter domains of the functional SNPs that influence the transcriptional activity of *MMP-1 *and *MMP-3 *between OSCC patients and healthy control groups. Multivariate analysis was effectively used to assess correlations among parameters such as OSCC, the genotypes, age and sex. In this report, we describe the role of functional SNP of the *MMP-1 *gene in susceptibility to OSCC and, as a remarkable finding; we discuss the crucial impact of the *MMP-1 *2G allele in the development of OSCC in younger individuals.

## Methods

### Study subjects

The tested cases were all unrelated native Japanese comprising 170 cases of OSCC (107 males, 63 females; average age 56.5 ± 13.9 years) who were histopathologically diagnosed as differentiated squamous cell carcinoma. The controls comprised 164 (104 males, 60 females) healthy subjects who did not have a history of malignant tumors and were frequency-matched to the cases by age (± 5 years; average age 51.5 ± 14.7 years). All OSCC subjects were patients who had been treated in the Dental Department of Niigata University Medical and Dental Hospital, Special Dental Care and Oral Surgery, Shinshu University Hospital, and Division of Oral Surgery, Nagaoka Red Cross Hospital.

Blood samples were taken after obtaining the patients' informed consent to participate in the study and processed anonymously. All cases were diagnosed histopathologically as OSCC. The study protocol was approved by the ethics committees of each institution.

### Genotyping of *MMP-1 *and *MMP-3*

5 ml of blood was obtained from the subjects and used as the source of peripheral blood lymphocytes. 0.2% NaCl was added for destruction of red blood cells, followed by addition of TNE buffer, 10% SDS and Proteinase K (MERCK Co., Darmstadt, Germany). After incubation at 58°C for more than six hours, genomic DNA was extracted by phenol-chloroform treatment and ethanol precipitation. The reaction was performed in a 25 μl volume made up of 70 ng genomic DNA, 2.25 μl of the specific forward/reverse primer (10 μM), 1 μl of TaqMan^® ^MGB probes (5 μM), 12.5 μl of TaqMan^® ^Universal Master Mix (Applied Biosystems, Foster City, CA, USA). PCR cycling conditions were 10 min of initial denaturation at 95°C followed by 35 cycles of 15 sec denaturation at 92°C and 1 min of one step annealing/extension at 60°C for *MMP-1 *or 62°C for *MMP-3 *(ABI Prism^® ^7900 HT Sequence Detection System, Applied Biosystems). Combination of the probe/primer used for the *MMP-1 *SNP were: Forward primer, 5'-TGCCACTTAGATGAGGAAATTGTAGT-3' and reverse primer, 5'-ACACTTTCCTCCCCTTATGGATTC-3', TaqMan^® ^MGB probes, FAM for 1G, 5'-ATAATTAGAAAGATATGACTTATC-3' and VIC for 2G, 5'-ATAATTAGAAAGGATATGACTTAT-3'; used for the *MMP-3 *SNP were: Forward primer, 5'-ACATCACTGCCACCACTCTGTT-3' and reverse primer, 5'-GGCACCTGGCCTAAAGACATT-3', TaqMan^® ^MGB probes, FAM for 5A, 5'-AAGACATGGTTTTTC-3' and VIC for 6A, 5'-AGACATGGTTTTTTC-3'.

### Statistical analysis

The Chi-square test was used to examine the differences in genotype distribution of the *MMP-1 *and *MMP-3 *promoters between the OSCC cases and control groups, and to estimate correlation or synergistic effects of *MMP-1 *genotypes with regard to clinical consequences as well as environmental factors among the OSCC cases. A *p*-value of < 0.05 was considered as statistically significant. Univariate analysis was performed by Fisher's exact test (two-sided) on gender, carrier state of *MMP-1 *1G allele (1G+ or 1G-) and 2G allele (2G+ or 2G-) between the OSCC and control groups. In the consideration of latent interrelations between factors, the effects of *MMP-1 *2G allele, age and gender were estimated by a multivariate logistic regression model. For each parameter, the OSCC risk was accounted by Odds Ratios (OR) and 95% Confidence Intervals (95% CI). All of the statistical analyses were performed using the SPSS 11.5J software package (SPSS Japan Inc., Tokyo, Japan).

## Results

### Genotype distributions of *MMP-1 *and *MMP-3 *promoter polymorphisms in OSCC cases and control groups

The genotype distributions of the promoter SNPs in *MMP-1 *and *MMP-3 *genes did not indicate a departure from the Hardy-Weinberg equilibrium when they were examined separately in the OSCC (n = 170) and control (n = 164) groups, or in all the samples combined (n = 334) (χ^2 ^< 3.84, *p *> 0.05). The frequency of 1G/2G or 2G/2G promoter genotypes having the 2G allele, which is associated with a high *MMP-1 *expression level, was significantly higher in the OSCC group, and the frequency of the 1G/1G homozygote was lower than that of the control group (*p *= 0.034, Table 1). On the other hand, no difference in *MMP-3 *genotype distribution (5A/5A, 5A/6A, and 6A/6A) was detected between the OSCC case and control groups.

**Table 1:** *MMP-1*and *MMP-3 *genotype distribution in OSCC cases and controls

	**OSCC cases **(*n *= 170)	**Controls **(*n *= 164)	***p***
***MMP-1***			
2G/2G	77 (45.3)	64 (39.0)	0.034
1G/2G	79 (46.5)	71 (43.3)	
1G/1G	14 (8.2)	29 (17.7)	
			
***MMP-3***			
5A/5A	3 (1.8)	8 (4.9)	0.188
5A/6A	50 (29.4)	54 (32.9)	
6A/6A	117 (68.8)	102 (62.2)	

The data were analyzed by the χ^2 ^test.

### Association between *MMP-1 *promoter genotypes and various clinical parameters

**Figure 1:**
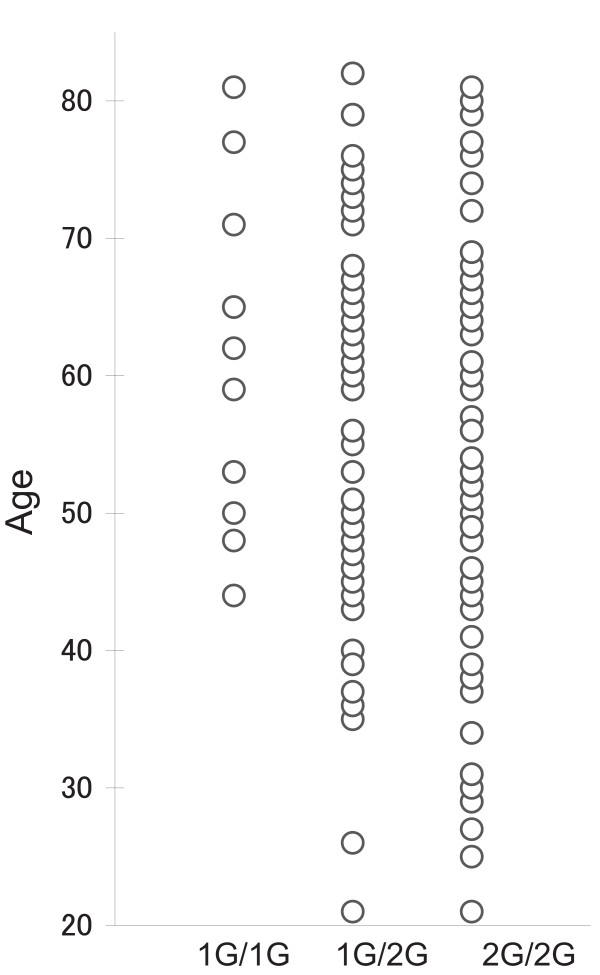
**Age distribution of the *MMP-1 *genotypes in 170 OSCC cases between the ages of 20 and 80 years**. The frequency of the 1G/1G genotype is notably low. Note the clear reduction of the 1G/1G and 1G/2G genotype distributions in individuals under the ages of 45 and 35 years, respectively.

*MMP-1 *promoter genotypes of OSCC cases were stratified by clinical parameters including gender, age (Table 2), T category, lymph-node metastasis (N category), tumor location in the oral cavity, status of alcohol intake and smoking (Table 3). A significant association was detected only between the age and *MMP-1 *genotype, which suggests accumulation of older age patients with the 1G allele, or young age patients with the 2G allele. Figure 1 shows the age distribution pattern of subjects with *MMP-1 *promoter genotypes in OSCC groups, demonstrating a remarkable reduction of the 1G/1G and 1G/2G genotype distributions in OSCC cases less than 45 years and 35 years old. Receiver operating characteristic (ROC) curve consistently exhibited that the 45 years of age could be an optimal cutoff value of the age at which to stratify the young OSCC from the older bracket in following analyses. It was of considerable interest to notice that the *MMP-1 *promoter genotype has a remarkable difference in distribution between subjects over and under 45 years of age. A significant difference was demonstrated in susceptibility to OSCC in individuals less or more than 45 years old (*p *= 0.002, OR = 2.26, 95%CI = 1.35–3.79) (Table 4). No association was observed with the 1G allele (1G/1G and 1G/2G; *p *= 0.294, OR = 1.29, 95%CI = 0.84–2.00, whereas a significantly elevated risk of OSCC was exhibited with the 2G allele (2G/2G and 1G/2G; *p *= 0.016, OR = 2.39, 95%CI = 1.21–4.72. The environmental factors, alcohol intake and smoking status were stratified with *MMP-1 *genotype distributions in OSCC cases, but no significant association was demonstrated of either factor with the *MMP-1 *genotype distribution (Table 3).

**Table 2:** Age and gender distribution in relation to alleles in cases and controls

	OSCC cases				Controls			
	2G/2G	1G/2G	1G/1G	*p*	2G/2G	1G/2G	1G/1G	*p*
Age*	54.8 ± 15.1	57.5 ± 13.2	60.1 ± 10.9	0.277	52.2 ± 14.8	52.3 ± 14.5	48.1 ± 14.2	0.387
**Gender^#^**								
*males*	46 (59.7%)	53 (67.1%)	8 (57.1%)	0.570	38 (59.4%)	46 (64.8%)	20 (69%)	0.640
*females*	31 (40.3%)	26 (32.9%)	6 (42.9%)		26 (40.6%)	25 (35.2%)	9 (31%)	

Data are expressed as mean ± SD or number of subjects (%).

* One-way ANOVA test.

^# ^χ^2 ^test.

**Table 3:** Relation of MMP-1 genotypes to clinical parameters and environmental factors among the OSCC cases

	**2G/2G ***n *(%)	**1G/2G ***n *(%)	**1G/1G ***n *(%)	***p***
**Total**	77 (45.3)	79 (46.5)	14 (8.2)	
				
**Gender**				
Male	46 (45.9)	53 (47.5)	8 (7.4)	0.57
Female	31 (47.2)	26 (41.3)	6 (7.5)	
				
**Age**				
(years ± SD)	54.8 ± 15.1	57.5 ± 13.2	60.1 ± 10.9	0.277*
				
**T category**				
T1-2	55 (45.1)	58 (47.5)	9 (7.4)	0.78
T3-4	22 (45.8)	21 (43.8)	5 (10.4)	
				
**N category**				
N0	50 (43.9)	53 (46.5)	11 (9.6)	0.607
N1-3	27 (48.2)	26 (46.4)	3 (5.4)	
				
**Tumor location**				
Tongue	45 (54.9)	31 (37.8)	6 (7.3)	0.260^#^
Lower gingiva	14 (35)	24 (60)	2 (5)	
Oral floor	6 (35.3)	9 (52.9)	2 (11.8)	
Buccal mucosa	3 (21.4)	9 (64.3)	2 (14.3)	
Upper gingiva	7 (53.8)	5 (38.5)	1 (7.7)	
Palate	2 (50)	1 (25)	1 (25)	
				
**Alcohol intake**				
Drinker	45 (45.9)	45 (45.9)	8 (8.2)	0.982
Non-drinker	32 (44.4)	34 (47.2)	6 (8.3)	
				
**Smoking status**				
Smoker	39 (44.3)	43 (48.8)	6 (6.8)	0.702
Non-smoker	38 (46.3)	36 (43.9)	8 (9.8)	

T and N categories were according to the UICC TNM classification of malignant tumors for lip and oral cavity. The data were analyzed by the χ^2^.

* One-way ANOVA test.

^# ^Fisher's exact tests.

**Table 4:** Univariate analysis of predictive factors

	**OSCC**	**Controls**	***p***	**OR (95% CI)**
**Age**			0.002	2.26 (1.35–3.79)
<45	29	52		
≧45	141	112		
**Gender**			1.000	0.98 (0.63–1.53)
**Male**	107	104		
**Female**	63	60		
**1G^*a*^**			0.294	1.29 (0.84–2.00)
**-**	77	64		
**+**	93	100		
**2G^*b*^**			0.016	2.39 (1.21–4.72)
**-**	14	29		
**+**	156	135		

***a***: 1G- denotes 2G/2G, and 1G+ denotes 1G/1G or 1G/2G. ***b***: 2G- denotes 1G/1G, and 2G+ denotes 2G/2G or 1G/2G.

Data were analyzed by Fisher's exact test. The odds ratio and 95% CI were calculated.

### Multivariate analyses of *MMP-1 *allele frequencies and clinical parameters

Logistic regression analysis of OSCC in relation to age, gender, and presence of the 1G and 2G alleles revealed that subjects of 45 years old and older had a 2.47-fold significantly increased risk of OSCC (*p *= 0.0006, 95%CI = 1.47–4.14) and that those carrying the *MMP-1 *2G allele had a 2.30-fold significantly elevated risk (*p *= 0.018, 95%CI = 1.15–4.58). Thus it was demonstrated that age and the presence of the 2G allele were independently associated with OSCC onset (Table 5).

**Table 5:** Multivariate analysis of factors affecting OSCC

**A **Results of factors affecting OSCC				
	***β *coefficient**	**OR**	**95% CI of OR**	***p***

**2G+**	0.83	2.30	(1.15–4.58)	0.0180
**Age**	0.90	2.47	(1.47–4.14)	0.0006
**B **Results showing the alleles independently associated with OSCC (adjusted for age and gender).				

**Allele**	***β *coefficient**	**OR**	**95% CI of OR**	***p***

**1G/2G**	0.782	2.187	(1.06–4.51)	0.034
**2G/2G**	0.907	2.476	(1.20–5.13)	0.015

**A**: 2G+ : 2G- = 0, 2G+ = 1

Age : <45 = 0, ≧45 = 1

**B**: With adjustment for age and gender Alleles are included as multinomial variables with categorical coding in reference to 1G/1G allele.

% concordance = 74.9%.

Hosmer-Lemeshow Goodness-of-fit test: χ^2 ^test = 0.996 ; *p *= 0.998

## Discussion

In this study, we examined SNPs in the promoter regions of *MMP-1 *and *MMP-3 *genes in relation to OSCC risk with case-control analyses. Significant differences, i.e., high frequency of 2G/2G genotype and decreased frequency of 1G/1G genotype, were observed in the *MMP-1 *genotype distribution in OSCC cases (*p *= 0.034, Table 1). Similarly, in comparisons of allele frequency a significant correlation was detected between onset of OSCC and genotypes that carry the *MMP-1 *promoter 2G allele in homo- or hetero-form (*p *= 0.016, OR = 2.39, 95%CI = 1.21–4.72). *MMP-1 *with the 2G type promoter caused a higher expression level of *MMP-1 *in tissues [11]. The biological mechanism of the elevated risk of OSCC as related to the 2G allele of *MMP-1 *promoter includes genetic instability, which is accompanied by the cell cycle activation caused by a sequence of events led by increased *MMP-1 *enzyme activity, i.e. activation of tissue disruption, reconstruction and resultant release of ECM binding growth factors. The relationship between *MMP-1 *promoter polymorphism and risk of OSCC has been investigated by Lin *et al. *in a group of 121 cases [22] and by Cao *et al. *in a group of 96 cases [23] and by Vairaktaris *et al*. in a group of 156 cases [24]. Although both Lin *et al. *and Cao *et al.*reported an elevation of OSCC risk correlated with the *MMP-1 *2G allele, further studies were needed to reach a conclusion mainly because the numbers of cases in the previous studies were relatively small, and there were differences between these reports in background analyses on correlation with environmental risk factors, such as smoking and areca use. In this study, significant correlations were found between the presence of the *MMP-1 *promoter 2G allele and increased OSCC risk among 170 Japanese OSCC patients. These results are consistent with existing reports on malignant neoplasms in general. It is notable that a new finding on the molecular function of *MMP-1 *in the onset of OSCC was obtained from the characteristic pattern of the *MMP-1 *promoter genotype distribution in younger patients.

Interestingly, Vairaktaris *et al. *reported an increased risk of oral cancer with MMP-1 1G/2G polymorphism [24]. The discrepancy of the results of the Chinese studies and our report on one hand and the European (German and Greek) study on the other hand may be explained by the diverse ethnic background of the different studied populations. Another point of difference is the fact that their patient sample included patients with positive family history of thrombophilia.

The observed bias of the average age by the *MMP-1 *promoter genotype among the 170 OSCC cases of this study suggested an influence of the *MMP-1 *promoter 2G allele on the age of OSCC onset. The scatter plot of age on the *MMP-1 *promoter genotypes also revealed disappearance of the 1G/1G-genotype distribution among the OSCC cases under 45 years old. In agreement with this observation, the ROC analysis suggested that the age of 45 years is the borderline age above which a change occurs in the incidence of OSCC. Although the specific reasons for these results have not been clarified, they suggest that some kind of biological conditions related to carcinogenesis have initiated around the age of 45. Carcinogenesis is caused by synergistic effects of various factors [25,26]. The multivariate logistic regression analysis showed that aging and presence of the *MMP-1 *2G allele (i.e., 2G/2G or 1G/2G) are independently involved in onset of OSCC. In fact, it was clarified that the significance of the presence of the *MMP-1 *2G allele was lower in the subject group 45 years old or older (*p *= 0.238, OR = 1.65, 95% CI = 0.75–3.55), and that the impact of the allele was highly significant in the younger group, which was under 45 years old (*p *= 0.014, OR = 9.67, 95% CI = 1.20–78.15) (data not shown).

As concrete causes of OSCC, mainly tobacco smoking and alcohol usage and, in some cases, viral infections have attracted attention as environmental causative factors [25,27]. It has been suggested that tobacco smoking and alcohol usage alone may not explain the mechanism of the entire early onset of OSCC, because the duration of exposure to these risk factors in young OSCC patients is shorter than in the older group, and also because there are some cases with stable incidence that have no known risk factors [28]. The association with smoking and habitual drinking has been supported as the risk factors of OSCC, which generally shows a higher incidence in older age, but other investigators believe that the mechanism of the early onset OSCC is fundamentally different from that of elderly onset OSCC [25]. Cao *et al. *considered the relationship with smoking as a behavioral risk factor and the *MMP-1 *2G allele as a genetic risk factor in the OSCC group [23]. In lung cancer, it has been similarly shown that a genetic factor of the *MMP-1 *promoter 2G allele increases the risk of cancer that occurs in a tobacco-usage-dependent manner only in cases with a history of tobacco use. These reports support the role of *MMP-1 *promoter polymorphism as an endogenous background factor, in contrast to exogenous environmental factors [15]. However, no evidence to support a notion of synergistic interactions between risk factors, such as habitual smoking and drinking and *MMP-1 *promoter polymorphism in OSCC cases, was shown in this study. Further investigations with regard to frequency and duration of exposure are required to consider interactions between environmental factors and multiple genetic background factors.

Examples are known of malignant neoplasms caused by genetic factors, for example, mutations in *RB *in Retinoblastoma [29], *p53 *in Li-Fraumeni Syndrome [30], and *APC *in familial adenomatous polyposis [31]. There are also reports of mutations in *BRCA1 *and *BRCA2*, which affect familial breast cancer occurrence, though their penetrance is low [32]. These are neoplasms that are all caused by loss-of-function mutations in molecules functionally classified as tumor-suppressor genes, and they are characterized by familial, juvenile or young onset and may be multicentric or bilateral. Although incidents are rare in the population, they are examples of germline mutations that directly involve heterofamilial tumorigenesis.

By contrast, the *MMP-1 *promoter 2G allele is a genetic polymorphism that exists at the high allele frequency of 80% to 90% in the general population. It is possible to call this a hereditary trait that is shared among races or humans in general. Other genetic factors that are classified into similar polymorphism as *MMP-1 *have also been reported, such as Cyclin D1 (*CCND1*) in head and neck squamous cell carcinoma (HNSCC) and colorectal cancer [33,34], xeroderma pigmentosum complementary group D (*XPD*), DNA damage binding protein 2 (*DDB2*), and *MMP-9 *in lung cancer [35-37]. Similar to the effects of mutations in tumor suppressor genes, diversity in gene expression level and molecular structure caused by genetic polymorphisms affect monitoring and repair mechanism of DNA replication, as well as control of the cell cycle, ultimately resulting in genetic instability. The common mechanism of carcinogenesis caused by those genetic factors is an elevated carcinogenic risk due to this genetic instability.

As examples of genetic polymorphisms that affect onset age of cancers other than that examined in this study, some reports discuss *MMP-1 *and *DDB2 *in lung cancer [15,36], and *CCND1 *in HNSCC and colorectal cancer [33,34]. These genetic factors, on the other hand, are present in patients with cancer as well as in the general population at certain frequencies. Therefore, it is hard to conclude that these genetic factors are the primary factors in early onset carcinogenesis, unlike germline single-gene mutations in *Rb *and *APC *that induce juvenile or early onset of cancers with high penetrance. Aging brings exposure to various carcinogenic factors and is associated with inevitable accumulation of genetic and epigenetic modifications of genes. These accumulations interact synergistically with biological background factors on the host side, and consequently cancer may develop [26,38]. It seems that inherited genetic factors play a larger role in development of early onset cancer that has undergone a relatively short duration of exposure to carcinogen through environment and lifestyle habits. Therefore, it is conceivable that the impact of these genetic factors would appear more directly in younger than in older cases, although we still expect a synergistic involvement of various environmental and genetic factors. Nevertheless, because of the disappearance ofthe *MMP-1 *1G allele distribution observed in the OSCC cases with a clear boundary under the age of 45 years, the *MMP-1 *promoter 2G allele should be recognized as an essential genetic precondition for the development of early onset OSCC.

It is said that an onset age of 45 years and younger accounts for about 6% of OSCC cases [27]. It should be noted that in this study we found that the tongue was the primary site in 25 cases (86.2%) of 29 early onset OSCC cases in individuals under 45 years old. This primary site was obviously different from that in patients 45 years old and older in whom we noted 57 cases of tongue cancer (40.4%) out of 141 OSCC cases. Similar tendencies have been reported in previous studies [39,40]. These findings suggest the possibility that each primary tumor sites in the oral cavity may be associated with differences in cancer susceptibility, specific carcinogenic stimulation, or genetic background. However, no environmental or genetic factors specific to a particular site of the oral mucosa have been identified for OSCC. It is conceivable that it would be important to carry out further investigations on genetic and environmental risk factors for each anatomical site of the oral regions, though we are not able to discuss this aspect of OSCC because the number of cases was inadequate in this study.

## Conclusion

The number of cases in this study was relatively small; therefore, the conclusions may still be contingent upon confirmation in a larger study. To evaluate the conclusive impact of *MMP-1 *on OSCC risk, a sufficiently large number of cases or consistency among results from multiple age adjusted studies would be needed. Furthermore, although the *MMP-1 *promoter SNP-based determination of OSCC susceptibility has the potential to become a useful clinical tool, it is necessary to add a greater diversity of information for practical use. In the future, improvements in diagnosis of susceptibility and clinical application could become possible by incorporating multiple genetic and environmental risk factors into the prediction model for cancer occurrence. Information about the risk of various types of malignant neoplasm as well as OSCC is useful for prevention and early detection of cancer or for monitoring of postoperative recurrence. A diagnostic system for evaluating cancer risk is vital not only for improvement in treatment techniques but also as a theoretical foundation to build strategies for cancer prevention.

## Abbreviations

Matrix metalloproteinase (MMP), single nucleotide polymorphism (SNP), oral squamous cell carcinoma (OSCC), odds ratio (OR), 95% Confidence Intervals (95% CI), receiver operating characteristic curve (ROC), head and neck squamous cell carcinoma (HNSCC).

## Competing interests

The author(s) declare that they have no competing interests.

## Authors' contributions

RN, MN participated in the design of the study. RN, MN and AAN drafted and wrote the manuscript. NK participated in the statistical analysis. RN, MN and AAN participated in the production of genotype data. RN, MN, KK, MO and HK participated in the acquisition and interpretation of data. RN, MI, HF, TK and AAN participated in the experimental studies. RN, MN, AAN, HH and SS carried out the clinical studies. SC, HY and RT participated in the review of the study. All authors read and approved the final manuscript.

## Pre-publication history

The pre-publication history for this paper can be accessed here:


